# Analysis of the Global Population Structure of *Paenibacillus larvae* and Outbreak Investigation of American Foulbrood Using a Stable wgMLST Scheme

**DOI:** 10.3389/fvets.2021.582677

**Published:** 2021-02-26

**Authors:** Bojan Papić, Margo Diricks, Darja Kušar

**Affiliations:** ^1^Institute of Microbiology and Parasitology, Veterinary Faculty, University of Ljubljana, Ljubljana, Slovenia; ^2^bioMérieux, Applied Maths NV, Sint-Martens-Latem, Belgium

**Keywords:** *Paenibacillus larvae*, American foulbrood, whole-genome sequencing, whole-genome multilocus sequence typing, core-genome multilocus sequence typing, whole-genome single nucleotide polymorphism

## Abstract

*Paenibacillus larvae* causes the American foulbrood (AFB), a highly contagious and devastating disease of honeybees. Whole-genome sequencing (WGS) has been increasingly used in bacterial pathogen typing, but rarely applied to study the epidemiology of *P. larvae*. To this end, we used 125 *P. larvae* genomes representative of a species-wide diversity to construct a stable whole-genome multilocus sequence typing (wgMLST) scheme consisting of 5745 loci. A total of 51 *P. larvae* isolates originating from AFB outbreaks in Slovenia were used to assess the epidemiological applicability of the developed wgMLST scheme. In addition, wgMLST was compared with the core-genome MLST (cgMLST) and whole-genome single nucleotide polymorphism (wgSNP) analyses. All three approaches successfully identified clusters of outbreak-associated strains, which were clearly separated from the epidemiologically unlinked isolates. High levels of backward comparability of WGS-based analyses with conventional typing methods (ERIC-PCR and MLST) were revealed; however, both conventional methods lacked sufficient discriminatory power to separate the outbreak clusters. The developed wgMLST scheme provides an improved understanding of the intra- and inter-outbreak genetic diversity of *P. larvae* and represents an important progress in unraveling the genomic epidemiology of this important honeybee pathogen.

## Introduction

*Paenibacillus larvae* is a Gram-positive bacterium that is the causative agent of American foulbrood (AFB), a devastating disease affecting honeybee (*Apis mellifera*) larvae (1). The disease is highly contagious through the extremely resistant *P. larvae* spores and causes considerable economic losses in the apiary industry throughout the world. The most effective method to prevent the spread of AFB disease is to burn the infected beehives and contaminated equipment (2). Alternatively, when AFB is in its primary stages or colonies show no signs of the disease, the shook swarm method can be applied to prevent disease onset (3).

Reproducible typing methods with sufficient discriminatory power are essential for the epidemiological surveillance of infectious diseases and outbreak investigations. Several genotyping methods have been applied to study the molecular epidemiology and population structure of *P. larvae*, including (*i*) the repetitive element PCR fingerprinting (rep-PCR), in particular the enterobacterial repetitive intergenic consensus-based polymerase chain reaction (ERIC-PCR) (4), (*ii*) pulsed-field gel electrophoresis (PFGE) (5), and (*iii*) multilocus sequence typing (MLST) (6). These conventional methods give roughly concordant results with regard to *P. larvae* distribution (i.e., clearly separate the epidemiologically most relevant types ERIC I and II), but with various degree of discriminatory power and repeatability (5, 6). For the past two decades, ERIC-PCR has been the most widely used method for *P. larvae* genotyping, classifying *P. larvae* into four ERIC types (ERIC I–IV) (5). Recently, a novel ERIC V type has been described (7). ERIC types differ in their phenotypic characteristics, including their virulence (5, 7–9).

The aforementioned conventional typing methods lack the discriminatory power needed to accurately delineate outbreak clusters. Thus, whole-genome sequencing (WGS) has recently become the method of choice for microbial typing due to its unprecedented discriminatory power and the ability to elucidate true phylogenetic relationships (10). The two most commonly used WGS-based approaches include the whole-genome single nucleotide polymorphism (wgSNP) analysis and the allele-based analysis (10, 11). In the SNP-based approach, the single-nucleotide changes are used to infer phylogenetic relatedness. In the allele-based approach, a non-redundant set of genes that are present across a set of genomes representing a species is compared in a gene-by-gene approach, known as the whole-genome MLST (wgMLST) approach. Alternatively, only the loci that are present in the majority (usually 95–99%) of isolates can be compared, known as the core-genome MLST (cgMLST) approach (12). To apply wg/cgMLST, an allele database for the population of a bacterial species must first be set. Such scheme can be constructed in a single-use *ad-hoc* manner and is typically based only on a specific subset of genomes (e.g., isolates from a single country or study). Alternatively, it can be constructed in a stable manner, based on a set of genomes representing the species-wide genetic diversity. *Ad-hoc* schemes use a local nomenclature, whereas the stable public schemes are subjected to appropriate curation and allow for a unified and expendable nomenclature, facilitating the exchange of results between laboratories.

Currently, WGS-based studies of *P. larvae* global population structure are lacking and only one study applying *ad-hoc* cgMLST for the outbreak investigation of AFB has been published (13). Thus, we developed a stable whole-genome MLST (wgMLST) scheme for *P. larvae*, which was based on a set of 125 genomes from 12 globally distributed countries and included all known ERIC types (I–V). The created scheme was used to assess the global population structure and the correlation between wgMLST and conventional typing methods (ERIC-PCR and MLST). In addition, wgMLST, cgMLST, and wgSNP were compared with respect to their suitability to delineate outbreak clusters, including those formed by 51 *P. larvae* isolates from Slovenia sequenced in this study.

## Materials and Methods

### Slovenian *P. larvae* Genome Dataset

**Figure 1:**
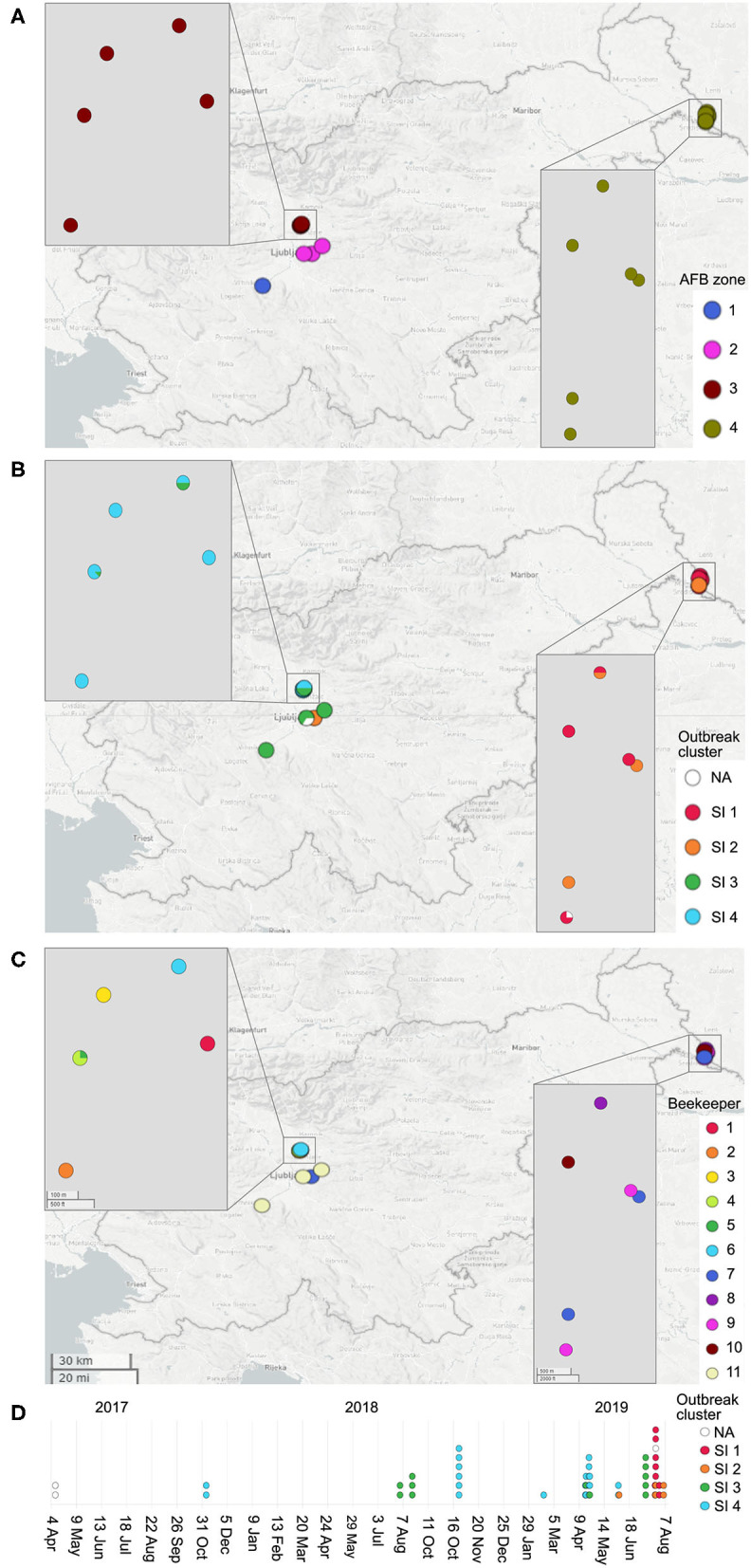
Map of the investigated AFB outbreak in Slovenia, 2017–2019. Circles denote the 18 apiaries from which 51 *Paenibacillus larvae* isolates were obtained. Circles are colored according to the AFB zone **(A)**, outbreak cluster **(B)** and beekeeper **(C)**. **(D)** denotes the timeline of the outbreak. Positions of the circles are defined by the geographic coordinates of the apiaries. In most cases, more than one isolate per apiary was obtained; thus, the circle size is not proportional to the number of isolates. Note that four apiaries in AFB zone 3 have an overlapping location. Three isolates (PL33, PL40, and PL41) did not cluster within any of the identified outbreak clusters (denoted as “NA”, not assigned). For additional isolate metadata, see Supplementary Table 1.

A total of 51 *P. larvae* isolates obtained in the 2017–2019 period during AFB outbreak investigation in Slovenia (SI) were sequenced and typed to assess the epidemiological applicability of the constructed wgMLST scheme. The isolates originated from an AFB outbreak covering four AFB zones, defined in Slovenia as a 3-km radius surrounding each AFB-positive apiary. The AFB zones were located in geographically distant regions of Slovenia, altogether encompassing 18 apiaries maintained by 11 beekeepers. In most cases, more than one isolate per apiary, but from different honeybee colonies, was obtained. AFB zones were epidemiologically linked through migratory beekeeping or beekeepers. The map showing the spatio-temporal distribution of outbreak-related isolates was constructed using Microreact (14) (Figure 1). For isolate metadata including apiary and colony origin, see Supplementary Table 1.

#### DNA Extraction

For the extraction of total genomic DNA from 51 *P. larvae* isolates of the SI dataset, pure culture stocks, stored at −80°C, were grown on blood agar plates at 37°C for 3 days. A loop-full of *P. larvae* culture was subjected to DNA extraction using the DNeasy Blood & Tissue Kit (Qiagen) following the manufacturer's protocol for Gram-positive bacteria. DNA was stored at −80°C prior to ERIC-PCR and WGS.

#### Whole-Genome Sequencing

The extracted DNA from 51 *P. larvae* isolates from the SI dataset was quantified using the Qubit 1 × dsDNA High-Sensitivity Assay Kit with the Qubit 3.0 fluorometer (both Thermo Fisher Scientific) and subjected to agarose gel electrophoresis to confirm its integrity. DNA was fragmented using the EpiSonic 2000 Sonication System (EpiGentek) and DNA libraries were constructed using the NEBNext Ultra DNA Sample Prep Master Mix Kit (NEB). The paired-end (2 × 150 bp) sequencing was performed on the NovaSeq 6000 System (Illumina) to a minimum coverage of 200 ×.

#### ERIC-PCR

For 51 *P. larvae* isolates from the SI dataset, ERIC-PCR was performed as previously described (4, 5) but optimized with respect to the primer concentration and selection of DNA polymerase. A 25-μl reaction contained 1 × Multiplex PCR Master Mix (Qiagen), 2 μM of each primer, and 5 μl of DNA template. For ERIC-PCR profile determination, the amplicons were analyzed by QIAxcel capillary electrophoresis using the QIAxcel DNA High Resolution Kit with QX Alignment Marker 15–5,000 bp and QX Size Marker 100–2,500 bp (all Qiagen). Separation was performed using the OM500 method and sample injection time of 10 s.

Since the assembly of short-read data is hampered by repetitive regions, short-read data do not enable the reconstruction of a closed chromosome and ERIC-PCR typing cannot be performed *in silico*. Thus, backward comparability of WGS and ERIC typing was assessed on a set of 143 isolates for which both WGS and ERIC-PCR typing results were available; the analysis was performed only for ERIC types I and II due to their epidemiological significance and sufficient number of isolates. Isolate SAG 10367/CP020557.1 was excluded from the analysis since its ERIC type is unclear (15, 16). The results of both methods were considered concordant if ERIC type corresponded with one of the two major phylogenetic clusters observed in the wgMLST tree corresponding to ERIC types I and II.

### Public *P. larvae* Genome Dataset

All the publicly available *P. larvae* complete genomes (*n* = 10), draft genomes (*n* = 4) and SRA data (*n* = 114) were downloaded from the NCBI database (http://www.ncbi.nlm.nih.gov/) on 17 February 2020, representing the public genome dataset.

Supplemented with 51 isolates from Slovenia, the complete genome dataset for the analysis of the global population structure consisted of 179 *P. larvae* genomes. In addition to WGS data, genome metadata were collected where available, which included, but were not limited to, the genome assembly quality metrics, year and country of isolation, outbreak association, MLST, and ERIC types (Supplementary Table 1). SRA data (including data from the SI dataset) were assembled *de novo* in the BioNumerics software v7.6.3 (Applied Maths NV, bioMérieux) using SPAdes v3.7.1 (17) and the resulting assemblies were annotated using Prokka v1.14 (18).

### *In silico* 7-Gene MLST

The developed wgMLST scheme also includes the seven MLST loci of *P. larvae* scheme (6) implemented within the PubMLST database (19). MLST sequence types (STs) were determined from the assembled genomes using BioNumerics. STs corresponded to the public nomenclature implemented within the PubMLST database. The Comparing Partitions online calculator (http://www.comparingpartitions.info) was used to calculate the Simpson's index of diversity (1–D), measuring the probability that two isolates randomly selected from a population belong to different MLST STs.

### wgMLST Scheme Creation

For the creation of a stable wgMLST scheme for *P. larvae* typing, five complete genomes (chromosomal and plasmid sequences) and 120 high-quality draft genome assemblies were used as input (i.e., the reference genome dataset) for the in-house scheme creation pipeline developed by Applied Maths. Of these 120 draft genomes, 47 were obtained in this study and represent a subset of the Slovenian *P. larvae* genome dataset (*n* = 51) passing the employed quality parameters. These genomes originated from at least 12 globally distributed countries and covered all ERIC (I–V) types, thereby representing a species-wide diversity of *P. larvae*. Detailed description of the scheme creation workflow and technical validation is given in Supplementary File 1.

### wg/cgMLST Typing

Determination of the allele number for each locus was performed in BioNumerics using two different allele calling algorithms: (*i*) the assembly-free (AF) allele calling, which uses a *k-*mer approach and starts from the raw sequence reads, and (*ii*) the assembly-based (AB) allele calling, which performs a BLASTn search against the assembled genomes with reference alleles of each locus as query sequences (Supplementary File 2). The results of AF and AB allele calling were then combined into a single set of allele assignments called summary calls (Supplementary File 1).

Pan-genome analysis was performed by determining the total number of loci present in random subsets of one up to 179 genomes. For each sample size, 100 random subsets were analyzed and the minimum, maximum, and average number of loci present was calculated. The core-genome for a given set of samples was calculated as the subset of wgMLST loci for which at least a certain percentage (90, 95, or 99%) of the genomes had an assigned allele number. Unless stated otherwise, the 95% core-genome of the complete dataset was used.

The wg/cgMLST trees were constructed in BioNumerics. Categorical coefficients were used for defining similarity levels and unweighted pair group method with arithmetic mean (UPGMA) was used as the clustering algorithm. The similarity between two samples was calculated as the number of loci with the same allele number divided by the number of loci two samples have in common. Topscore UPGMA tree was defined as the UPGMA tree with the highest resampling support calculated from 200 permutations. The wgMLST tree of the complete genome dataset was visualized and annotated using iTol v4.4.2 (20). Comparison of the cgMLST and wgMLST trees was performed using the tanglegram algorithm implemented in Dendroscope v3.7.2 (21).

The minimum spanning trees (MSTs) were constructed in BioNumerics using the wg/cgMLST allele profiles as input data. The pairwise distance matrix was calculated by counting the number of pairwise allele/SNP differences. Only loci for which both of the compared genomes had a valid allele call (i.e., loci with an assigned allele number) or SNP were taken into account. To compare the genetic diversity of ERIC I and ERIC II based on wgMLST, the median, minimum, and maximum pairwise wgMLST allele differences were also calculated, taking into account only a single isolate per wgMLST cluster (single-linkage distance of ≤35 alleles, as defined in the paragraph Outbreak Cluster Delineation).

### wgSNP Typing

Reference-based wgSNP analysis was performed in BioNumerics. SRA data were first downsampled to 100 × coverage where applicable. Raw reads were mapped using Bowtie v2.2.5 (22) by applying the parameters '-a, -p 8 -mm' using GCA_002951875.1 (CP019651.1) and GCA_002951895.1 (CP019652–4.1) as reference genomes for ERIC I and ERIC II, respectively. In the resulting consensus sequence, a position was considered to be unreliable if the coverage was below the threshold or behaved abnormally (i.e., showed significant local coverage drop compared with the surrounding bases). The minimum total, forward and reverse coverage were set to 3, 1, and 1, respectively. The single, double and triple base thresholds were 75, 85, and 95%, respectively. If more than 50% of reads contained a deletion, a gap was introduced. The strict SNP filtering template was applied to retain relevant, high-quality SNPs. This filter removes positions with at least one unreliable, ambiguous base (non-ACTG), or gap as well as non-informative SNP positions (i.e., positions that contain SNPs relative to the reference sequence but where all sequences under study have the same base). Each of the retained SNPs had a minimum coverage of 5 × and was present at least once in both forward and reverse direction. The minimum distance between the retained SNPs was 12 bp. The maximum-likelihood phylogeny was inferred using RAxML v8.2.12 (23) with the following parameters: “-f a -x 12345 -p 12345 -# autoMRE -m GTRGAMMA”. Phylogenetic trees were visualized and annotated using iTol v4.4.2 (20).

## Results

### Creation and Technical Validation of wgMLST Scheme

A set of 125 high-quality publicly available *P. larvae* genomes was used as input for wgMLST scheme creation (Supplementary Table 1, Supplementary File 1). The final wgMLST scheme consisted of 5738 wgMLST loci (complete open reading frames, ORFs; Supplementary Table 2) and seven loci from the conventional MLST scheme (partial ORFs) described previously (6) and is commercially available through a plugin in BioNumerics. The complete genome dataset (*n* = 179) was then used to assess the global population structure and elucidate the core-genome and pan-genome of *P. larvae*.

### Core-Genome and Pan-Genome Analysis

Each genome had an average of 3809 wgMLST loci with an assigned allele number. ERIC I genomes had a larger pan-genome compared with ERIC II genomes (4767 and 3981 loci, respectively), mainly because of the higher average number of accessory loci per genome (Table 1). The core, i.e., genes present in at least 95% of the genomes of the complete dataset (*n* = 179), consisted of 2833 loci. Nonetheless, clustering of the samples based on only this set of core loci resulted in a nearly identical tree topology as compared with wgMLST analysis (Supplementary Figure 4 in Supplementary File 1).

**Table 1:** *Paenibacillus larvae* core-genome and pan-genome analysis.

**ERIC type**	**No. of genomes**	**No. of wgMLST loci ± SD**	**No. of accessory loci (95%) ± SD**	**No. of loci in the pan-genome**	**No. of core loci (99%)**	**No. of core loci (95%)**	**No. of core loci (90%)**
ERIC I	89	3,982 ± 124	544 ± 90	4,767	2,097	3,460	3,640
ERIC II	80	3,614 ± 37	228 ± 30	3,981	2,903	3,396	3,483
ERIC I–V	179	3,809 ± 209	999 ± 202	5,738	1,951	2,833	3,177

*The core loci were calculated as the subset of wgMLST loci present in at least 90, 95, and 99% of the genomes under study, respectively. Only loci with a valid allele call (i.e., loci with an assigned allele number) were taken into consideration. The average number of accessory loci per genome was calculated based on the core loci present in at least 95% of the genomes under study*.

*SD, standard deviation*.

**Figure 2:**
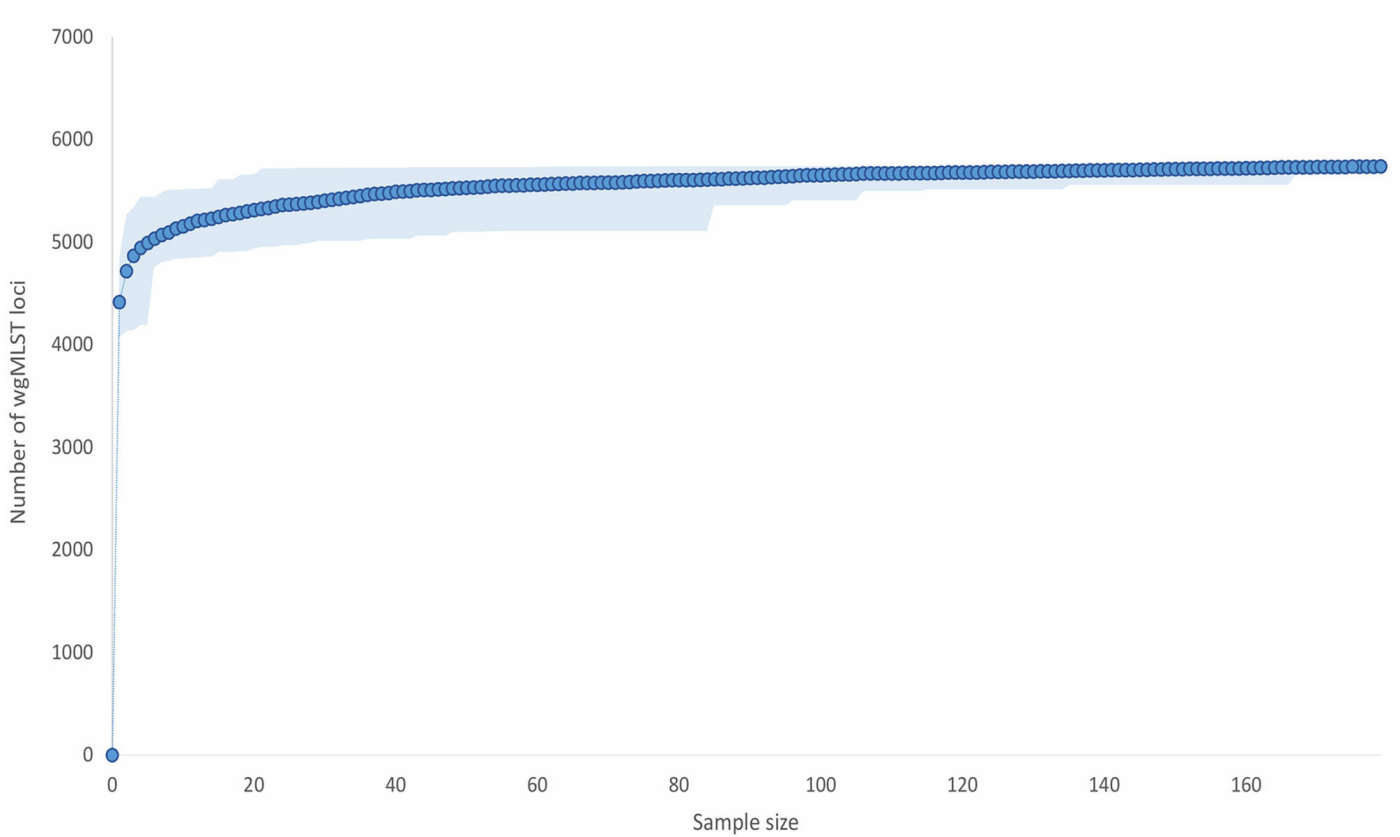
*Paenibacillus larvae* pan-genome gene accumulation curve. For each sample size (x-axis), 100 repeats of random selections of genomes from the complete dataset (*n* = 179) were performed and the average, minimum, and maximum number of loci present were calculated. The present loci included all the detected loci (>80% identity to the reference allele(s) of the corresponding loci), i.e., both valid and invalid (ambiguous bases, no start/stop codon, internal stop codon, or <85% identity) allele calls were taken into account. The average number of the present loci is displayed as full lines, whereas the minimum and maximum numbers represent the limits of the light-colored zones.

*Paenibacillus larvae* pan-genome accumulation curve quickly approached the asymptote, which is indicative of a relatively closed pan-genome (Figure 2). A sample size of 21 genomes encompassed >99% of the total number of wgMLST loci (*n* = 5,738).

### Population Structure Analysis

#### ERIC Type

**Figure 3:**
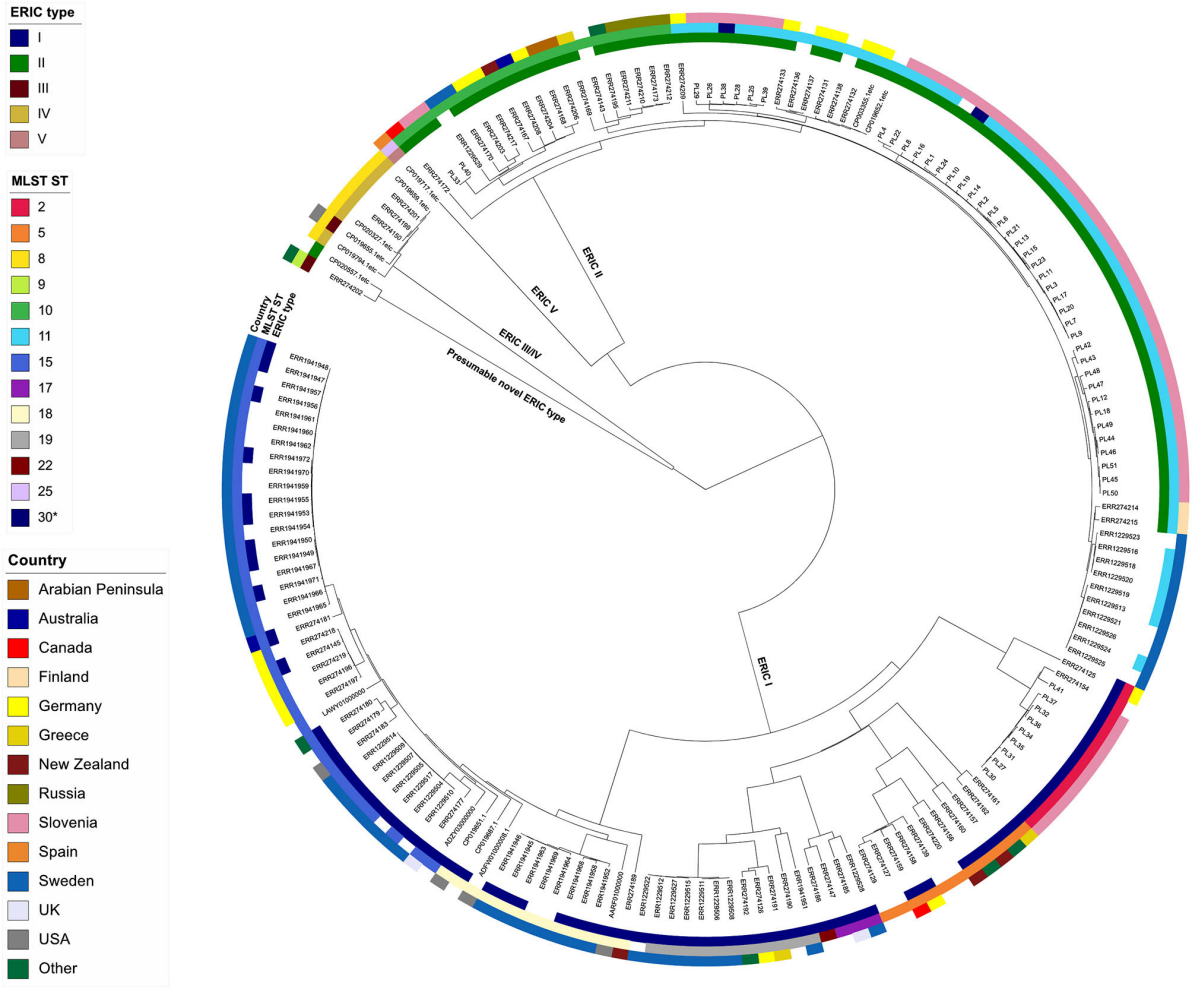
wgMLST tree of the complete *Paenibacillus larvae* genome dataset (*n* = 179). The tree was produced in BioNumerics using the categorical (values) similarity coefficient and UPGMA clustering algorithm and was annotated with the results of conventional typing methods (ERIC-PCR and MLST) and country of origin using iTol. Missing data are denoted as empty boxes.

The wgMLST tree revealed five major clades, generally corresponding to different ERIC types (Figure 3). Of the 143 isolates with an assigned ERIC type, the majority belonged to ERIC I (67/143; 46.5%) and ERIC II (66/143; 46.2%, excluding the isolate SAG 10367) types, which also formed two genetically distant and clearly separated wgMLST clades (Figure 3, Supplementary Table 1). ERIC I encompassed a larger number of STs (*n* = 7) as compared with ERIC II (*n* = 3). Simpson's index of diversity (1–D), calculated based on MLST STs, was higher in ERIC I (0.807; 95% confidence interval (CI): 0.761–0.853) compared with ERIC II (0.437; 95% CI: 0.328–0.544). The median pairwise wgMLST allele distance was 931 (range: 41–1,143) in ERIC I and 175 (range: 57–255) in ERIC II. Taken together, the genetic diversity was higher in ERIC I compared with ERIC II, as has already been reported previously based on MLST analysis (6). Two strains (11-8051/ERR274202 and SAG 10367/CP020557) that were representative of a presumable novel ERIC type formed a distinct wgMLST clade (Figure 3) and were excluded from the assessment of congruence between WGS phylogeny and ERIC type since their ERIC type is unclear (16, 24). ERIC III and IV types formed a common wgMLST clade (ERIC III/IV cluster) and were also excluded from the analysis of concordance between WGS phylogeny and ERIC type. The concordance between the observed wgMLST clades and ERIC type was 100% for both ERIC I and II.

#### MLST Type

The 170 isolates with an unambiguously assigned MLST type belonged to 13 different STs, of which two SI isolates (PL24 and PL38) constituted a novel ST, which was defined as ST30 by the *P. larvae* PubMLST database. A novel *ftsA* variant characteristic of ST30 was amplified by PCR as described previously (6) and confirmed by Sanger sequencing. ST11 (54/170; 31.8%) was the predominant MLST type, followed by ST15 (36/170; 21.2%) and ST10 (19/170; 11.2%). In total, 8/9 isolates without an assigned ST had ambiguous bases in the *Natrans* gene (*n* = 7) or a missing *Natrans* gene (*n* = 1) (Supplementary Table 1). The absence of *Natrans* gene in the SI isolate PL1 was confirmed by conventional PCR according to the protocol described by Morrissey et al. (6). The strain SAG 10367 (NCBI accession number CP020557.1) initially did not have an assigned ST (Supplementary Table 1), but clustered with the ST9 isolate (11-8051/ERR274202) on the wgMLST tree (Figure 3). In addition, indel errors are a known limitation of PacBio data; thus, the two deletions in the *Natrans* and *glpF* genes can be ignored, implying that the isolate belongs to ST9.

The concordance between the major clades observed in the wgMLST tree (corresponding to ERIC types) and MLST ST was 100% (i.e., all STs had a corresponding ERIC type) (Figure 3). MLST and wgMLST trees revealed a similar topology with minimum intertwining; however, wgMLST provided a much higher discriminatory power compared with MLST since several wgMLST clusters were observed within a single MLST ST; this was evident both in ERIC I and ERIC II genomes (Figure 3, Supplementary Figure 5 in Supplementary File 1). Two notable discordances between wgMLST clusters and MLST STs were observed: (*i*) one ST22 isolate clustered among ST17 isolates, and (*ii*) two ST30 isolates clustered among ST11 isolates (Figure 3). Of note, ST designation of these isolates was based on *in silico* analysis, except for ST30 isolates, which were confirmed by conventional MLST. Conventional MLST results were available for 47 isolates (Supplementary Table 1) and coincided in 46/47 (97.8%) cases with the results of *in silico* MLST.

### Outbreak Cluster Delineation

#### SI Outbreak Clusters

To assess the epidemiological applicability of WGS for outbreak cluster delineation, 51 *P. larvae* isolates obtained during epidemiological investigation of AFB outbreak in four geographically distant AFB zones in Slovenia in the 2017–2019 period were typed in this study using ERIC-PCR and three different WGS-based approaches, namely cgMLST, wgMLST, and wgSNP. Genetically closely related non-outbreak strains from the complete genome dataset were added to the analysis to enable the establishment of a threshold with an optimal sensitivity to detect outbreaks and specificity to exclude non-outbreak isolates.

**Figure 4:**
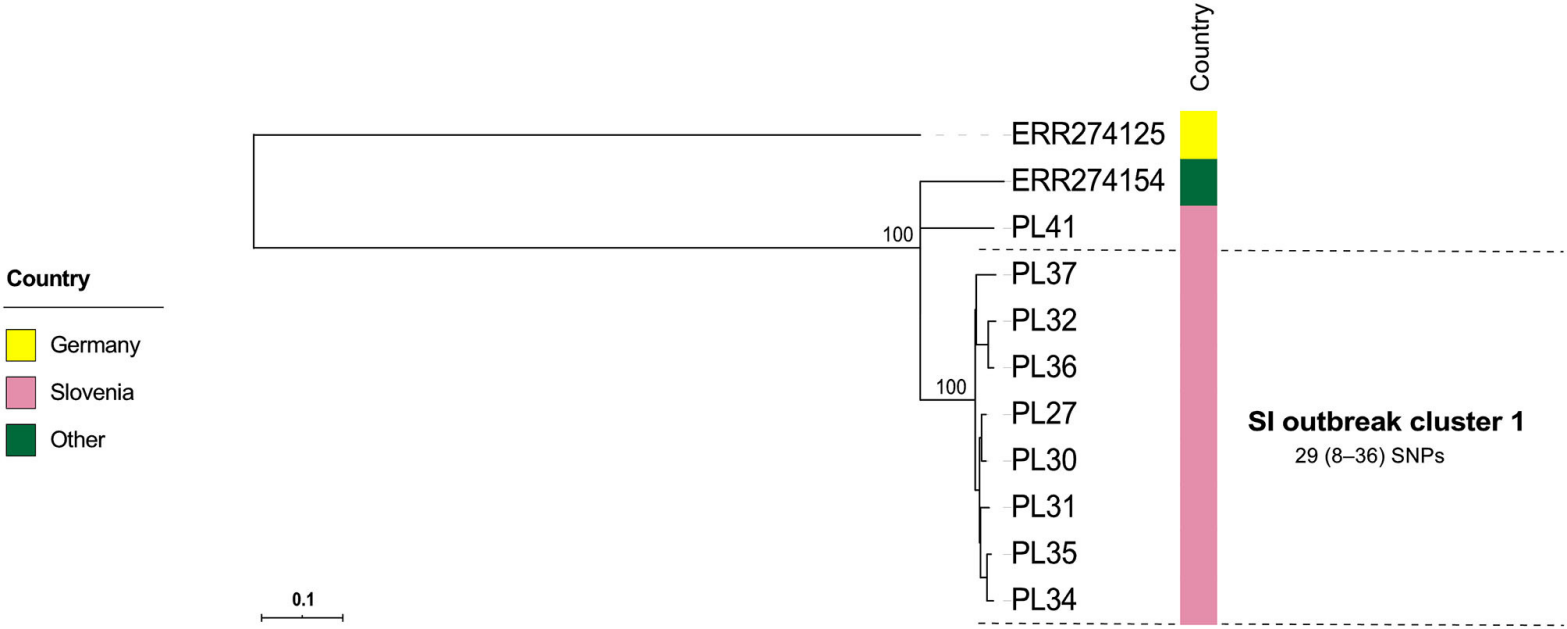
Maximum-likelihood phylogenetic tree based on genome-wide SNPs of the Slovenian outbreak-related *Paenibacillus larvae* isolates of the ST2-ERIC I type. Genetically most closely related non-outbreak isolates were included in the analysis. The identified outbreak clusters are colored and labeled according to the country of origin. The wgSNP analysis was performed in BioNumerics and phylogenetic tree was inferred using RAxML. Numbers above the branches denote bootstrap values; bootstrap values for the levels below the denoted outbreak clusters have been omitted for clarity, but were all 100%. Missing data are denoted as empty boxes. Scale bar indicates the average number of nucleotide substitutions per site.

**Figure 5:**
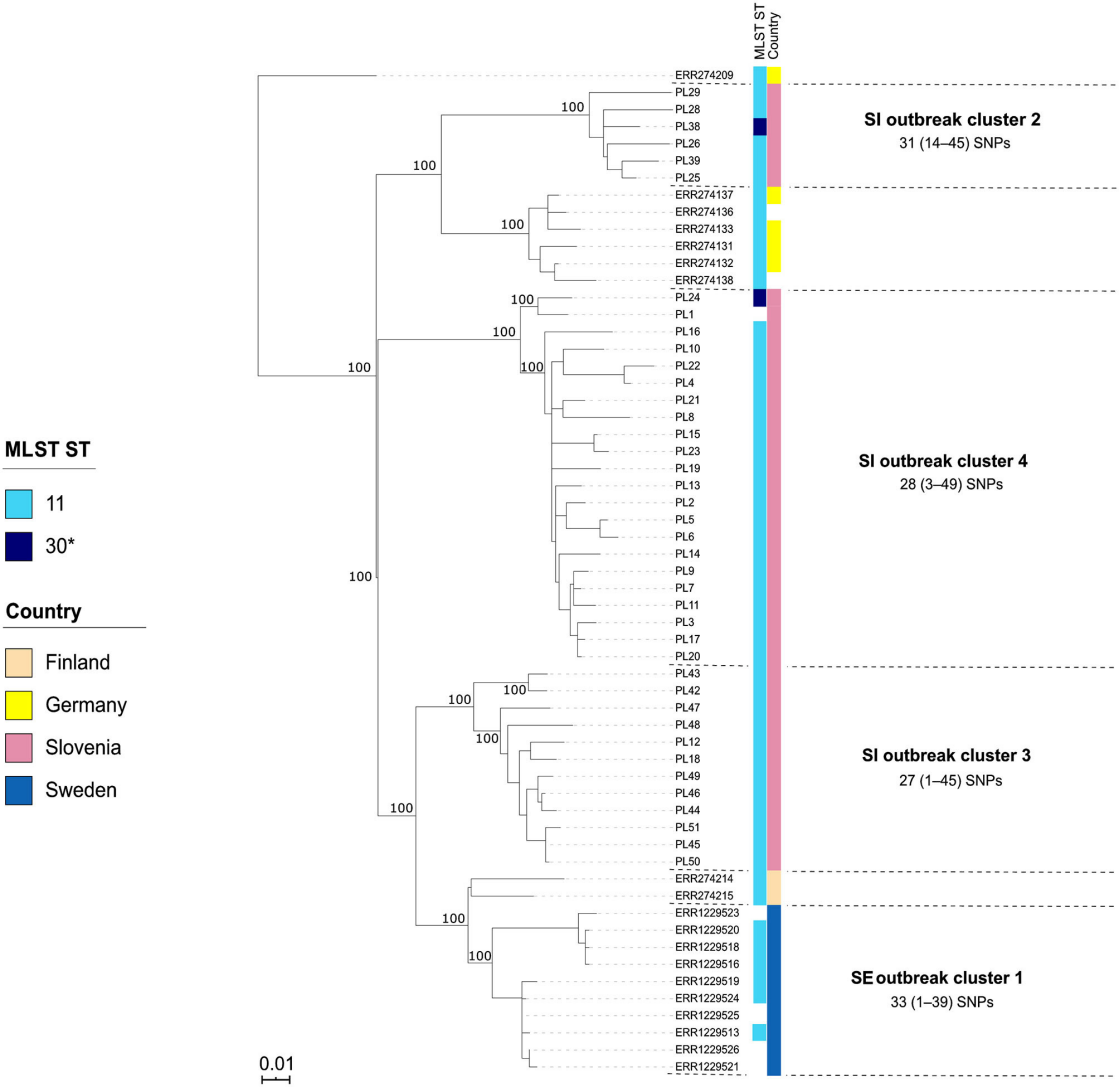
Maximum-likelihood phylogenetic tree based on genome-wide SNPs of the Slovenian (SI) outbreak-related *Paenibacillus larvae* isolates of the ST11/30-ERIC II type. Genetically most closely related non-outbreak isolates were included in the analysis. The identified outbreak clusters are colored and labeled according to the country of origin. The wgSNP analysis was performed in BioNumerics and phylogenetic tree was inferred using RAxML. Numbers above the branches denote bootstrap values; bootstrap values for the levels below the denoted outbreak clusters have been omitted for clarity, but were all 100%. Missing data are shown as empty boxes. SI outbreak cluster 3 and SE outbreak cluster 1 consisted of two genetically closely related (single-linkage distance of ≤35 alleles) groups (subclusters) that were epidemiologically linked and were thus interpreted as a single outbreak cluster, respectively. Of note, two SI isolates (PL24 and PL38) were of a novel ST30. Scale bar indicates the average number of nucleotide substitutions per site.

**Figure 6:**
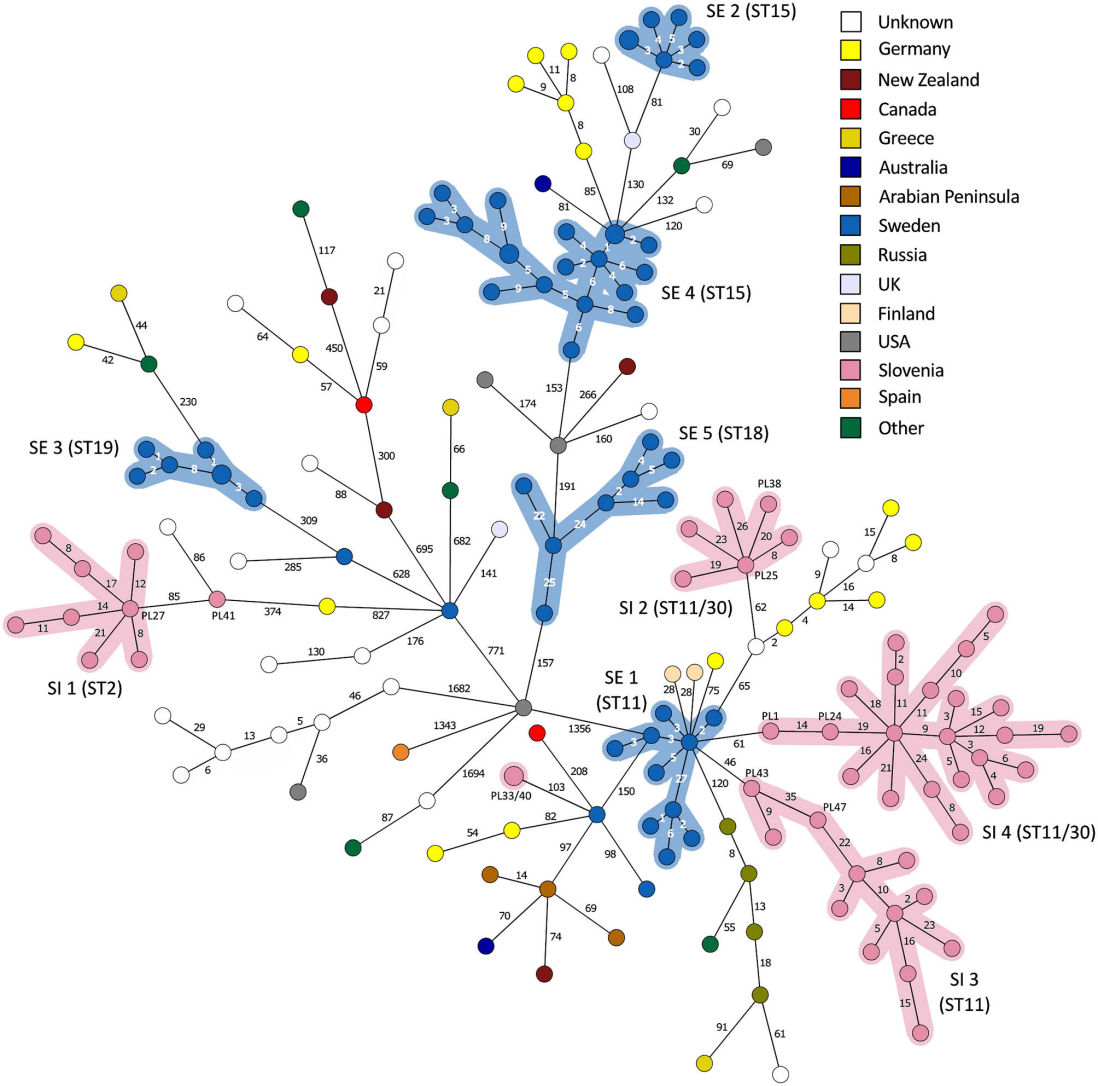
Minimum spanning tree based on 5738 wgMLST loci of the complete *Paenibacillus larvae* genome dataset (*n* = 179). Branch lengths are scaled logarithmically and branch labels indicate the number of allele differences. Nodes are colored according to the country of origin. Clusters of outbreak-related isolates with a single-linkage distance of ≤35 alleles are highlighted. Of note, two ST30 isolates from Slovenia clustered within the SI outbreak clusters 2 (isolate PL38) and 4 (isolate PL24), respectively.

The majority (42/51; 82.4%) of SI isolates belonged to ERIC II, whereas the remaining nine (17.6%) isolates belonged to ERIC I (Supplementary File 3). The wgMLST analysis revealed one ST2-ERIC I outbreak cluster (designated “SI outbreak cluster 1”) and three ST11-ERIC II outbreak clusters (designated “SI outbreak clusters 2–4”), which were further investigated by cgMLST and wgSNP (Figures 4–6, Table 2). The intra-outbreak diversity of ERIC I and ERIC II SI outbreak clones was comparable (Table 2). Two outbreak clones (SI outbreak clones 2 and 3) occurred in more than one AFB zone, suggesting their transmission between geographically distant (>3 km) apiaries. In three out of four AFB zones, two outbreak clones were observed, suggesting their co-circulation within a single AFB zone (Figure 1). Of note, two SI isolates (PL24 and PL38) that clustered within the SI outbreak clusters 4 and 2, respectively, were of a novel ST30 (Figures 5, 6). The intermixing of different STs within a single outbreak cluster (as defined by WGS) points out the limitations of conventional MLST in cluster analysis.

**Table 2:** Intra-outbreak diversity based on the pairwise genetic differences among the outbreak-related *Paenibacillus larvae* isolates from Slovenia (SI) and Sweden (SE).

**Outbreak cluster**	**MLST ST-ERIC type**	**No. of isolates**	**Median (min–max) cgMLST allele difference**	**Median (min–max) wgMLST allele difference**	**Median (min–max) wgSNP distance**
SI outbreak cluster 1	ST2-ERIC I	8	18 (8-24)	19 (8–28)	29 (8–36)
SI outbreak cluster 2	ST11-ERIC II (isolate PL38 was of ST30)	6	24 (8–31)	25 (8–31)	31 (14–45)
SI outbreak cluster 3	ST11-ERIC II	12	21 (1–36)	23 (2–40)	27 (1–45)
SI outbreak cluster 4	ST11-ERIC II (isolate PL24 was of ST30)	22	20 (2–37)	22 (2–38)	28 (3–49)
SE outbreak cluster 1	ST11-ERIC II	10	25 (1–28)	27 (1–30)	33 (1–39)

*See Figures 4–6 for additional information*.

Contrary to wg/cgMLST, wgSNP enables a construction of phylogenetic tree with confidence values (e.g., bootstrap support) and may provide additional discriminatory power. Thus, all outbreak clusters identified by wgMLST were further investigated by wgSNP. All SI outbreak clusters showed monophyletic clustering and a high (100%) bootstrap support in the genome-wide SNP phylogenetic trees (Figures 4, 5). The minimum, median and maximum SNP/allele distances of all the identified clusters are shown in Table 2. The minimum inter-outbreak genetic distance between the identified SI outbreak clusters of the ST11-ERIC II type was 69 wgMLST alleles and 92 SNPs. When compared with the complete genome dataset, the minimum genetic distance between the SI outbreak-related and non-outbreak isolates from other countries was 46 wgMLST alleles and 60 SNPs. In all SI outbreak clusters, the epidemiologically unrelated but genetically closely related strains were clearly separated from the outbreak clusters on the SNP phylogenetic trees (Figures 4, 5). Three SI isolates (PL33, PL40, and PL41) did not cluster within the identified SI outbreak clusters and were thus genetically unrelated although spatially related to the identified outbreak clones. Because the isolates PL33 and PL40 were genetically identical but originated from two different apiaries (maintained by two different beekeepers), AFB zones and isolation years (obtained in 2019 from brood and 2017 from honey, respectively), this may indicate circulation and/or transmission of a clone with potential to cause AFB after several years of persistence in honeybee environment. This shows the ability of WGS to reliably exclude the genetically unrelated but epidemiologically related strains from the outbreak. Comparison of the wgMLST and wgSNP approaches revealed a highly concordant clustering with minimal intertwining within the identified ST2 and ST11 outbreak clusters (Supplementary Figures 6, 7 in Supplementary File 1).

The epidemiological data supported all the identified SI outbreak clusters (Figure 1). The clusters generally corresponded with the AFB zones that were geographically distant, suggesting a local spread of *P. larvae* clones between the apiaries that are located within the same AFB zone (Figures 1A,B). Moreover, in several cases, the apiaries were maintained by the same beekeeper (Figure 1C). Three cases of transmission of the outbreak clone between different AFB zones were observed, which could be explained by the associated epidemiological data. The transmission of the outbreak clone SI 2 between AFB zones 2 and 4 could be explained by a common beekeeper (Figure 1C). The transmission of the outbreak clone SI 3 between AFB zones 1, 2, and 3 could be explained by a common beekeeper and/or migratory beekeeping (i.e., movements of mobile apiaries to a common foraging site) (Figure 1C).

On the MSTs built from wg/cgMLST allele distance matrices, all SI outbreak clusters could be identified using a single-linkage distance of 35 and 24 alleles, respectively. Hence, the maximum single-linkage distance within a cluster in the wgMLST MST was 35 wgMLST loci (Figure 6). The highest intra-outbreak diversity was observed in SI outbreak cluster 3, in which the isolates PL42 and PL43 formed a separate subcluster and differed from the remaining isolates by 35 wgMLST alleles (Figure 6). These two isolates were epidemiologically linked by a common beekeeper and originated from the same AFB zone. In addition, they were most closely related to the remaining isolates from this cluster; thus, they were considered part of the same outbreak cluster (Figure 5).

#### Other Outbreak Clusters (Public Genome Dataset)

By applying the same single-linkage threshold of 35 wgMLST alleles to the complete genome dataset, five additional outbreak clusters associated with a previously described AFB outbreak in Sweden [SE; (13)] were identified (Figure 6). In addition, two clusters associated with a presumable outbreak in Germany (8, 25) were observed, but were not interpreted as AFB outbreaks due to the lack of extensive epidemiological data. Co-circulation of several phylogenetically diverse lineages was observed within a single country (Figures 3, 6). When outbreak-related isolates were excluded from MST, no clear phylogeographic clustering was observed when considering the global population of *P. larvae*, as previously reported (6).

Although the isolates belonging to the SE 1 and SI 2–4 outbreak clusters were all of ST11-ERIC II type, except for two ST30 isolates clustering among the SI outbreak clusters 2 and 4, the WGS analysis clearly separated the clusters (Figures 5, 6). When analyzing WGS data from the previously described AFB outbreak in Sweden (13), we identified five SE outbreak clusters, of which one (SE 1) consisted of two groups of closely related and epidemiologically linked isolates (subclusters) separated by 27 wgMLST alleles (Figure 6). Two epidemiologically unrelated isolates from Finland differed in only 28 wgMLST alleles from this outbreak cluster but were excluded from the cluster due to lack of epidemiological link. This clearly shows that thresholds should not be interpreted as fixed values but rather as a guide in establishing potential links between isolates and considered in combination with extensive epidemiological data.

For the previously described AFB outbreak in the Upland region (13), two outbreak clusters were identified (SE outbreak clusters 4 and 5, belonging to ST15-ERIC I and ST18-ERIC I, respectively) (Figure 5). The Gotland ST15-ERIC I cluster was clearly separated from the Upland ST15-ERIC I cluster by >200 wgMLST allele differences (SE outbreak clusters 2 and 4, respectively; Figure 5). The minimum, median, and maximum pairwise distances within the identified SE outbreak clusters (Figure 5) were very similar to those previously reported (13). Moreover, the definitions of the outbreak clusters presented herein are the same as those previously established if the two closely related groups (subclusters) of the SE outbreak cluster 1 are considered part of the same cluster (Figures 4, 5).

## Discussion

Reliable typing methods with sufficient discriminatory power are an integral part of the surveillance of infectious diseases. Here, we applied WGS to study the molecular epidemiology of *P. larvae* on a set of 179 genomes representative of a species-wide diversity. In the first part of the study, we developed a stable wgMLST scheme and assessed the backward comparability of WGS-based phylogeny with conventional typing methods (MLST and ERIC-PCR). In the second part of the study, we confirmed the epidemiological applicability of the constructed wgMLST scheme on a set of 51 *P. larvae* isolates obtained within the framework of AFB outbreak investigation in Slovenia and previously described AFB outbreaks in Sweden (13).

### Population Structure Analysis

WGS-based phylogeny revealed five major *P. larvae* clades, generally corresponding to different ERIC types. The majority (133/143; 93.0%) of the analyzed genomes with an assigned ERIC type corresponded to the two epidemiologically most important ERIC types (ERIC I and II), which formed two clearly separate and genetically distant wgMLST clades (Figure 3). Our findings suggest that ERIC I and II types can be reliably predicted from WGS data based on mapping of the query genome against a reference dataset of genomes with a known ERIC type. For the 51 isolates from Slovenia analyzed in this study, WGS-based prediction of ERIC type corresponded perfectly with the results of conventional ERIC-PCR typing, supporting this hypothesis (data not shown). However, additional studies are needed to confirm the observed concordance between WGS and ERIC-PCR on a large and diverse panel of isolates typed by conventional ERIC-PCR.

The other three major wgMLST clades corresponded to ERIC III/IV, ERIC V, and a presumable novel ERIC type. Our results confirmed previous MLST-based findings that ERIC III and IV types form a common wgMLST cluster and that the two types cannot be reliably separated according to MLST or cg/wgMLST phylogeny due to their genetic homogeneity (6, 7). However, these types are not epidemiologically relevant since they are only represented by historical strains (2). WGS-based phylogeny also recapitulates the recently described ERIC V as a novel type since its representative isolate was genetically distant from other ERIC types (7). Whole-genome MLST provided a considerable improvement to MLST with regard to discriminatory power and phylogenetic signal (Supplementary Figure 5 in Supplementary File 1).

The clade representing the putative novel ERIC type is shown in Figure 3, Supplementary Figure 5 in Supplementary File 1. This clade formed a separate clade which was genetically very distant from the remaining ERIC clades and was represented by the strains SAG 10367 (presumable ST9) and 11-8051 (ST9). Strain SAG 10367 has been reclassified from ERIC III (15) to ERIC II (16) because its ERIC pattern best matched (but was not identical to) that of ERIC II. Strain 11-8051 has been classified as ERIC III/IV (24), but is genetically closely related to the strain SAG 10367 both by MLST and wgMLST (this study). Moreover, the ERIC pattern of strain 11-8051 was different from those of the remaining ERIC III (strain LMG 16252) and ERIC IV (strain DSM 3615) strains (24). Genersch et al. (5) classified the strains LMG 16252 and DSM 3615 into two separate ERIC types, whereas Ebeling et al. (24) categorized these strains as ERIC III/IV type according to MLST analysis. ERIC-PCR is known to exhibit limited reproducibility and inter-laboratory comparability (26). Based on the high genetic distance between this wgMLST clade and the remaining clades as well as the uncertainty regarding the ERIC types of both strains, the present data suggest this wgMLST clade may represent a novel ERIC type (or more than one ERIC type, as observed in ERIC III/IV clade) and should thus be further investigated and characterized.

Contrary to ERIC-PCR, MLST is a sequence-based typing method and can be reliably determined *in silico* from WGS data. WGS-based *in silico* MLST has already widely replaced conventional (Sanger sequencing-based) MLST and can be extended to wg/cgMLST (10). In the present study, we were able to successfully type the majority (95.5%) of strains using *in silico* MLST and the identified STs correlated perfectly (100%) with the major clades identified by wgMLST (i.e., a single ST was attributed to only one ERIC type). Moreover, a high agreement between the *in silico* predicted (this study) and previously reported conventional MLST STs (6) was observed. In summary, the present results suggest WGS offers a reliable backward comparability with large historical data based on both conventional methods for typing of *P. larvae* (MLST and ERIC-PCR), in addition to providing a much higher discriminatory power and additional (phylo)genomic information needed for outbreak investigations.

### Outbreak Cluster Delineation

MLST and wgMLST phylogeny were generally concordant, confirming the previously described MLST-based population structure analysis and association between ERIC type and MLST ST (6, 7). However, in several cases, the identified outbreak clusters belonged to the same MLST-ERIC type. This confirms that none of the conventional methods employed in this study (ERIC-PCR and MLST) provided sufficient discriminatory power to accurately delineate the observed outbreak clusters. On the contrary, WGS clearly distinguished between outbreak-related and non-related (but genetically closely related) isolates, as has already been reported in AFB outbreak in Sweden (13). Additionally, intermixing of different STs within a single outbreak cluster (as defined by WGS) were observed, pointing out the limitation of conventional MLST in AFB outbreak investigation. On the contrary, all three WGS-based approaches proved suitable for epidemiological surveillance of *P. larvae* and successfully differentiated between outbreak-related and non-outbreak isolates. The maximum single-linkage distance within the SI outbreak clusters 1, 2, and 4 was 35 wgMLST and 24 cgMLST alleles (as determined by the wg/cgMLST MST analysis). The maximum single-linkage distance within the SE outbreak clusters (27 wgMLST and 20 cgMLST alleles) is also comparable to that reported previously (13) (26 alleles), who used an *ad-hoc*, ERIC type-specific cgMLST scheme constructed using the Ridom SeqSphere^+^ software.

Cluster identification should allow for some genetic diversity between the outbreak-associated isolates, but only to the extent that they can still be assumed to originate from the same source (27). Although we propose here the single-linkage threshold of 35 alleles to define the outbreak clusters, it should be noted that such allele/SNP thresholds should serve as a guide in aiding outbreak investigations rather than a fixed rule and should be re-assessed in future studies of AFB outbreaks.

Thresholds for cluster identification should be species- and case-specific and should account for genetic characteristics and inherent genetic diversity of bacterial (sub)populations in different sources (27, 28). In this study, the maximum pairwise genetic diversity within a single beekeeping practice was 40 wgMLST alleles (data not shown), which should be considered when establishing the relatedness threshold. The proposed 35-allele threshold is comparable to the genetic diversity observed in source attribution studies of foodborne outbreaks caused by *Escherichia coli, Salmonella enterica* and *Listeria monocytogenes*, in which the genetic diversity of the outbreak-related isolates from different sources was assessed (27, 29). Of note, the proposed 35-allele threshold was based on the wgMLST MST analysis and does not equal the maximum pairwise distance between the outbreak-related isolates, which tends to be higher (Table 2). Another factor that may influence the relatedness threshold is sampling bias (i.e., which representatives of bacterial subpopulations are sampled and analyzed), as discussed previously (27). For example, if additional sampling of the SI outbreak cluster 3 revealed novel representatives clustering between the two major subclusters (separated by 35 alleles in Figure 6), this would decrease the single linkage-based threshold.

Several studies have emphasized the role of interpreting the genetic relatedness in the context of phylogenetic clustering with genetically closely related but epidemiologically unrelated isolates (27, 28, 30). In this study, such isolates were added to the wgSNP analysis and generally clustered to the exclusion of the outbreak-related isolates. In addition, ultimate confirmation of the outbreak should always be based on extensive epidemiological data confirming the epidemiological linkage (27). Finally, technical errors may be introduced during isolate manipulation, library preparation, sequencing, or bioinformatics analysis, affecting the results of cluster analysis (31).

The wgMLST and cgMLST approaches exhibited a similar discriminatory power and showed a perfectly concordant topology, suggesting that both methods are appropriate for outbreak cluster delineation. This suggests that both approaches can be used for early detection of presumable outbreak clusters using a simple clustering algorithm based on UPGMA or MST method. In addition, standardized nomenclature allows for a rapid and easy inter-laboratory exchange of results. The outbreak clusters identified by wg/cgMLST can be further investigated by wgSNP, as suggested previously for other pathogens (32, 33). Contrary to allele-based approaches, wgSNP can provide additional discriminatory power and enable reconstruction of a statistically supported phylogenetic tree (33). On the other hand, wgSNP requires a high-quality and closely related reference template and the obtained SNP pairwise distances depend on the subset of genomes under study. Maximum-likelihood SNP phylogeny of Slovenian and Swedish isolates revealed all outbreak-associated isolates clustered monophyletically at a high (100%) bootstrap support, further confirming their linkage.

Pan-genome analysis revealed that the wgMLST scheme constructed from the complete *P. larvae* genome dataset is a good representation of the species' complete pan-genome. We confirmed previous findings that ERIC I pan-genome is larger than ERIC II pan-genome; the number of loci in the pan-genome was comparable to the number of protein-coding genes in ERIC I and ERIC II genomes reported previously (7, 34). Moreover, the size of 95% core genome in ERIC I and ERIC II genomes reported herein was comparable to that reported previously (13).

Because evolutionary changes cause diversification of a single bacterial strain into genetically diverse subpopulations, limited sampling may not retrieve a representative from the subpopulation that was involved in the transmission of AFB but a closely related one (27). In the present study, several *P. larvae* isolates from a single apiary and from a single beekeeping practice were collected and typed to mitigate this limitation. The wgMLST approach revealed closely related (single-linkage distance of ≤35 alleles) *P. larvae* subpopulations obtained from a single apiary or different apiaries of the same beekeeping practice (beekeeper). The highest genetic diversity of outbreak-related isolates (as determined by wgMLST) was observed in SI outbreak clusters 3 and 4, which also had the highest number of analyzed isolates, suggesting that the intra-outbreak genetic diversity increases with the number of analyzed isolates. Moreover, two outbreak clones, belonging to two different ERIC types, were observed in one apiary, indicating polyclonal outbreaks and complex pathways of disease transmission, which have already been observed previously (13).

*Paenibacillus larvae* spores can be transmitted both horizontally and vertically. In the horizontal mode of transmission, spores spread between the individuals within and between honeybee colonies; this can be a result of honeybee activities (robbing and drifting) or human activities (e.g., honeybee trade beekeeping activities and equipment) (2, 35). In the vertical mode of transmission, spores spread from a mother colony to a daughter swarm (2). WGS is an essential component of studying pathways of disease transmission. Here, we confirmed the transmission of several *P. larvae* outbreak clones between different honeybee colonies of the same apiary and between different apiaries belonging to the same outbreak. Moreover, we observed two cases of transmission between geographically distant regions in Slovenia, which could be explained by beekeeping activities (same beekeeper) or migratory beekeeping during the foraging season.

Our results did not reveal any clear phylogeographic pattern of *P. larvae* on a global level, which is in accordance with previous findings (6). Nonetheless, this hypothesis needs to be further confirmed on a larger dataset due to the relatively low number of currently available *P. larvae* genomes.

Although WGS is already considered a gold standard typing method for global surveillance of important food-borne pathogens such as *Listeria monocytogenes, Salmonella enterica* and *Escherichia coli*, some practical issues in implementing WGS into routine workflows remain. Different WGS-based bioinformatics workflows and approaches can arrive to different conclusions regarding outbreak cluster delineation and pairwise distance estimation and there is currently a lack of harmonized bioinformatics workflows for data analysis and exchange (36, 37). To develop a harmonized and easy-to-use pipeline for WGS typing of *P. larvae*, we constructed a stable wgMLST scheme, which is implemented in the BioNumerics software. The wgMLST approach applied herein provides common allele nomenclature and direct comparability of results by different laboratories and is thus suitable for global routine surveillance of *P. larvae*. In addition, the clusters identified by wg/cgMLST can be further investigated by wgSNP in BioNumerics using a user-defined complete or draft reference genome(s).

In conclusion, we showed that all three WGS-based approaches (cgMLST, wgMLST, and wgSNP) are suitable for epidemiological investigation of *P. larvae* and provide sufficient discriminatory power for outbreak cluster delineation. Thus, WGS should become one of the standard typing methods for epidemiological surveillance of *P. larvae*. In addition, WGS enables a good backward comparability with conventional typing methods (MLST and ERIC-PCR). The developed wgMLST scheme will facilitate the implementation of WGS typing of *P. larvae* and its harmonization between different laboratories. Moreover, this study improves our understanding of the inter- and intra-outbreak diversity of *P. larvae*, guiding future AFB investigations.

## Data Availability Statement

Publicly available datasets were analyzed in this study, which can be found in the NCBI Short Read Archive (SRA) database (https://www.ncbi.nlm.nih.gov/sra/) under accession numbers listed in the Supplementary Table 1. Sequence reads of the 51 *Paenibacillus larvae* isolates that were sequenced in this study were submitted to the SRA database under the study accession number PRJNA613377.

## Author Contributions

BP, MD, and DK: conceptualization and writing—review and editing. BP and MD: methodology/detailing the study, construction of public dataset, bioinformatics analyses and visualization, and writing—original draft. DK: funding acquisition. BP and DK: construction and sequencing of SI dataset. MD: scheme creation and technical validation. All authors: revised the manuscript and provided comments.

## Conflict of Interest

MD was employed at bioMérieux at the time of data analysis and drafting of the manuscript and hence has a business implication in this work. BioMérieux is a company designing, developing, and selling infectious disease diagnostics including the BioNumerics software. The remaining authors declare that the research was conducted in the absence of any commercial or financial relationships that could be construed as a potential conflict of interest.

## Abbreviations

AB: assembly-based
AF: assembly-free
AFB: American foulbrood
cgMLST: core-genome multilocus sequence typing
CI: confidence interval
ERIC-PCR: enterobacterial repetitive intergenic consensus-based polymerase chain reaction
MLST: multilocus sequence typing
MST: minimum spanning tree
ORF: open reading frame
PFGE: pulsed-field gel electrophoresis
rep-PCR: repetitive element PCR fingerprinting
SE: Sweden
SI: Slovenia
SNP: single nucleotide polymorphism
ST: sequence type
UPGMA: unweighted pair group method with arithmetic mean
wgMLST: whole-genome multilocus sequence typing
WGS: whole-genome sequencing
wgSNP: whole-genome single nucleotide polymorphism.
