# Supplementary material for: Analysis of the Global Population Structure of Paenibacillus larvae and Outbreak Investigation of American Foulbrood Using a Stable wgMLST Scheme
Source: Front Vet Sci. 2021 Feb 26;8:582677. doi: 10.3389/fvets.2021.582677 (PMC7952629; doi:10.3389/fvets.2021.582677)
Supplement: Supplementary file 3 [file Data_Sheet_1.PDF]

# *Supplementary Material*

**Supplementary File S1 associated with Papić *et al.* – Analysis of the global population structure of *Paenibacillus larvae* and outbreak investigation of American foulbrood using a stable wgMLST scheme**

**Description of the *Paenibacillus larvae* wgMLST scheme creation and evaluation**

## 1 wgMLST scheme creation

In total, 125 *Paenibacillus larvae* genomes were used as input for the in-house developed whole-genome multilocus sequence typing (wgMLST) scheme creation pipeline developed by Applied Maths. This set is hereinafter referred to as the *reference genome dataset*, for which the corresponding metadata and quality parameters can be found in Supplementary Table S1**.** The reference dataset consisted of five publicly available complete genomes (one for each ERIC type, including sequences of extrachromosomal elements where available) and 120 NCBI Sequence Read Archive (SRA) data that were assembled *de novo* using SPAdes v3.7.1 implemented in the BioNumerics software v7.6.3 (Applied Maths NV, bioMérieux). The SRA data had an average quality of at least 33 and all assemblies passed the following quality criteria: coverage of >50 ×, number of contigs <350, $N_{50}$ >24 kb and total contig length between 3.6 and 5.2 Mb.

The 125 isolates from the reference dataset originated from 12 globally distributed countries (Supplementary Figure S1). Of these, 47 isolates that were typed within the framework of this study originated from Slovenia. The reference dataset consisted of ERIC I ($n$ = 50), ERIC II ($n$ = 70), ERIC III ($n$ = 2), ERIC IV ($n$ = 2) and ERIC V ($n$ = 1) genomes and thus represented a species-wide and global representation of *P. larvae* genomes, which increased the robustness and the versatility of the developed wgMLST scheme. Assemblies were annotated using Prokka v1.14.

The scheme creation pipeline initially started from the complete set of annotated coding sequences (CDSs) extracted from the reference dataset. Within this set, loci that were subsets of each other or that overlapped were merged and rarely occurring loci or loci with a high ratio of unhealthy alleles were omitted. An unhealthy allele was defined as a sequence without a valid start/stop codon (ATG, CTG, TTG or GTG), internal stop codon (TAG, TAA or TGA) or non-ACTG bases. In addition, loci containing large tandem repeat areas were removed. Finally, multi-copy loci were removed so that 90% of the input genomes had less than 10 multi-copy loci.

To determine the allele number(s) corresponding to a unique allele sequence for each locus, two different algorithms were employed: (*i*) the assembly-free (AF) allele calling, which uses a *k*-mer approach (*k*-mer size of 35 and minimum coverage of 3) and starts from the raw sequence reads, and (*ii*) the assembly-based (AB) allele calling, which performs a BLASTn search against assembled genomes with the reference alleles for each loci as query sequences. The word size for the gapped BLAST search was set at 11 and only hits with a minimum homology of 80% were retained. After each round of allele identification, all the available data from the two algorithms (AF and AB) were combined into a single set of allele assignments called summary calls. If both algorithms returned one or multiple allele calls for a given locus, the summary call was defined as

the allele(s) that was assigned by both algorithms. If there was no overlap, no allele number was assigned for this particular locus. If for a specific locus, the allele call was only available for one algorithm, this allele call was included. If multiple allele sequences were found for a summary locus, only the lowest allele number was retained.

New sequences that were not yet present in the allele database after scheme creation were assigned a new allele number only if the sequence had a valid start/stop codon, had no ambiguous bases or internal stop codons, had at least 85% identity compared with one of the reference allele sequences and had no more than 999 gaps in the pairwise sequence alignment with the closest allele sequence from the same locus. These criteria are also set as default auto-submission criteria for each BioNumerics user working with the scheme.

**2 Technical validation of the created wgMLST scheme**

The scheme creation pipeline produced an initial wgMLST scheme constituting 5752 loci (5745 wgMLST loci and seven loci from the conventional MLST scheme) (https://pubmlst.org/plarvae/). In total, 337 loci were known to originate from extrachromosomal elements. Validation of the created wgMLST scheme was performed using the 179 publicly available *P. larvae* genomes (hereinafter referred to as the *complete genome dataset*), which consisted of 165 *de novo* assembled draft genomes, 10 complete genomes and four pre-assembled draft genomes, including the 125 genomes that were used for wgMLST scheme creation (*reference genome dataset*; Supplementary Table S1).

Scheme validation consisted of several steps. First, SRA data of the 51 isolates from Slovenia were assembled using BioNumerics (SPAdes v3.7.1 with default parameters and without downsampling) and Shovill v1.0.9 (https://github.com/tseemann/shovill) by applying the default parameters (SPAdes v3.13.1 and downsampling to 100× coverage) and their wgMLST AB allele profiles were compared for each isolate. In general, the Shovill assemblies did not have any ambiguous bases, were ~30 kb longer and had more loci (average = 16 loci) compared with the BioNumerics/SPAdes assemblies. On average, 26 loci per genome were polymorphic when the wgMLST results from Shovill and BioNumerics assemblies were compared. However, only two loci (PLAR_1177 and PLAR_814) had a true allele number difference between the two assemblies in three cases, whereas the remaining loci were polymorphic due to a missing allele call in one of the assemblies. When calculating pairwise similarity or distance matrices in BioNumerics, only the loci that were assigned an allele number in both genomes are taken into account during pairwise comparison. Therefore, only the loci with true allele number discrepancies (PLAR_1177 and PLAR_814) were removed from the scheme.

Next, the genome with SRA run accession number ERR1941951 was assembled three times and subsequently analyzed using the AF and AB algorithms. The wgMLST profile of these three replicates was identical except for one loci (PLAR_2105), which was removed from the scheme.

Lastly, the 5752 AB reference sequences with allele number 1 were concatenated and the resulting assembly was analyzed. Four loci were not identified (PLAR_3384, PLAR_3471, PLAR_4542 and PLAR_4509) and were thus removed from the scheme, whereas all the remaining loci were assigned allele number 1 as expected.

In summary, seven problematic loci were removed from the initial scheme after validation, resulting in a final scheme containing 5738 wgMLST loci and seven MLST loci (Supplementary Table S2). The created wgMLST scheme is commercially available through a plugin in BioNumerics.

The majority of the loci (96%) from the scheme had <10 different allele variants (Supplementary Figure S2). Only 10 (0.1%) loci had a sequence length exceeding 5000 bp. For the 165 SRA genomes, most of the AF calls coincided with the AB calls (line 'AB call(s) == AF call(s)' in Supplementary Figure S3). In total, 34 discrepancies were found in 13 loci between AB and AF allele calls in 29 out of 165 SRA genomes (column 'Nr.ConsensusUnknown' in Supplementary Table S1; lines 'AF call <> AB call', 'AB call not in AF calls' and 'AF call not in AB calls' in Supplementary Figure S3). The discrepancy was caused either by a different allele number or by the exclusion of the AF call from the AB calls or vice versa. Of note, discrepant allele calls do not result in a consensus allele call and are thus not taken into account when performing pairwise comparisons.

In some cases, the AF algorithm did not yield an allele number, whereas the AB algorithm did (Supplementary Figure S3: line 'AF absent/unknown and AB call(s)'), and vice versa (Supplementary Figure S3: line 'AF call(s) and AB absent/unknown').

Note that new valid alleles (i.e., alleles with ≥85% identity to one of the reference alleles of that locus, with a start/stop codon, no internal stop codons and no ambiguous bases) will only be submitted to the allele database when the AB algorithm was run. In summary, these results show that running both the AB and AF algorithm is advised to obtain the most reliable results.

## 3 Congruence of wgMLST with other analytical approaches

Tree topologies generated by different analytical approaches were compared by tanglegram analysis. wgMLST was compared with cgMLST (Supplementary Figure S4) and MLST (Supplementary Figure S5) on the complete genome dataset ($n$ = 179). wgMLST and wgSNP were compared on subsets of ST2-ERIC I isolates (Supplementary Figure S6) and ST11/30-ERIC II isolates (Supplementary Figure S7).

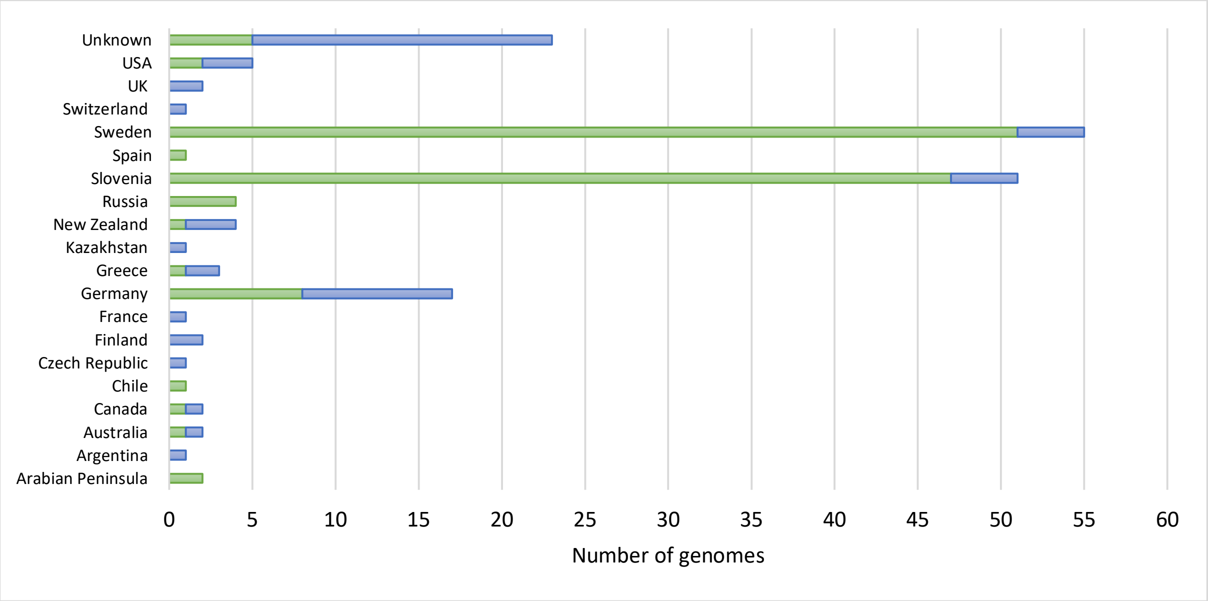


**Supplementary Figure S1.** Distribution of *Paenibacillus larvae* genomes from the complete dataset ($n$ = 179) per country of origin. The complete genome dataset was used for wgMLST scheme validation and population structure analysis. Green part represents the fraction of genomes that belonged to the reference dataset ($n$ = 125) used for the scheme creation, whereas the blue part represents the remaining genomes.

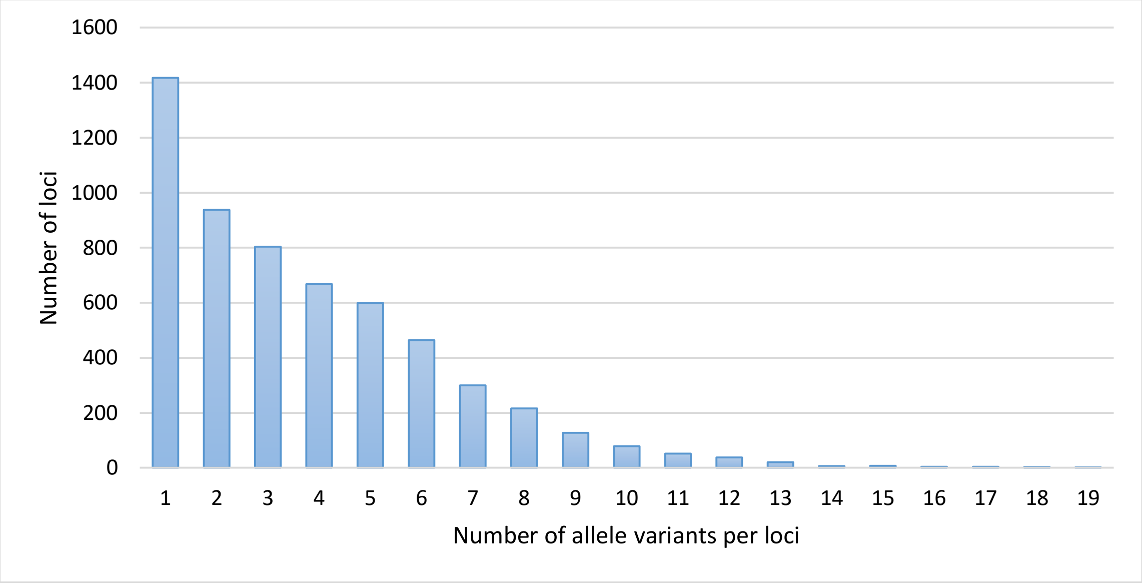


**Supplementary Figure S2.** Histogram of the number of allele variants per locus.

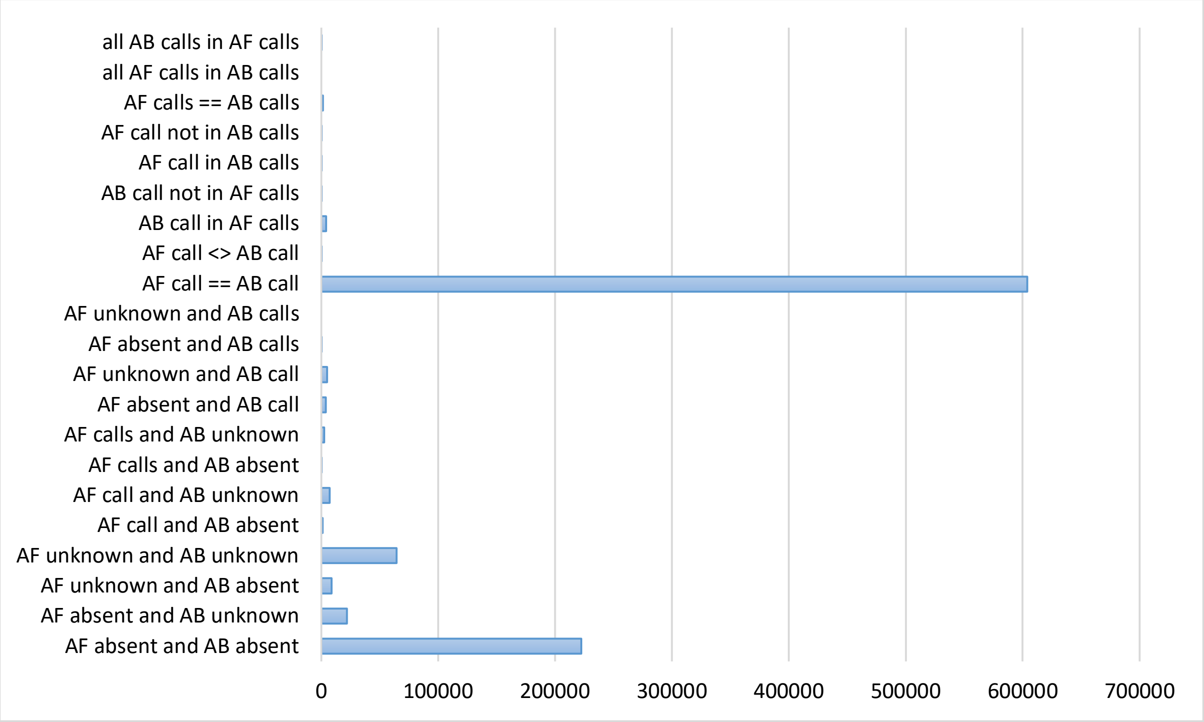


**Supplementary Figure S3.** Distribution of wgMLST allele call types for the *Paenibacillus larvae* SRA dataset ($n$ = 165).

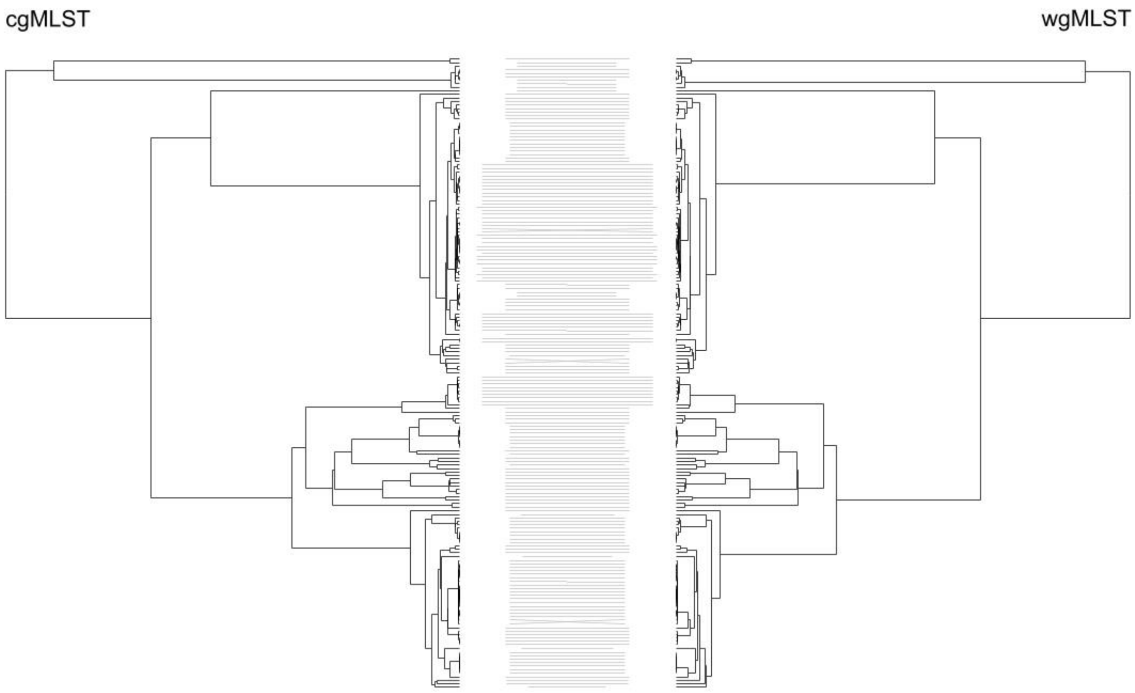


**Supplementary Figure S4.** Comparison of cgMLST and wgMLST. Tanglegram of cgMLST and wgMLST trees was constructed from the complete *Paenibacillus larvae* genome dataset ($n$ = 179) in Dendroscope. Both trees were produced in BioNumerics using the categorical (values) similarity coefficient and UPGMA clustering algorithm. Core genome was defined as a subset of wgMLST loci with an assigned allele number in at least 95% of the genomes under study.

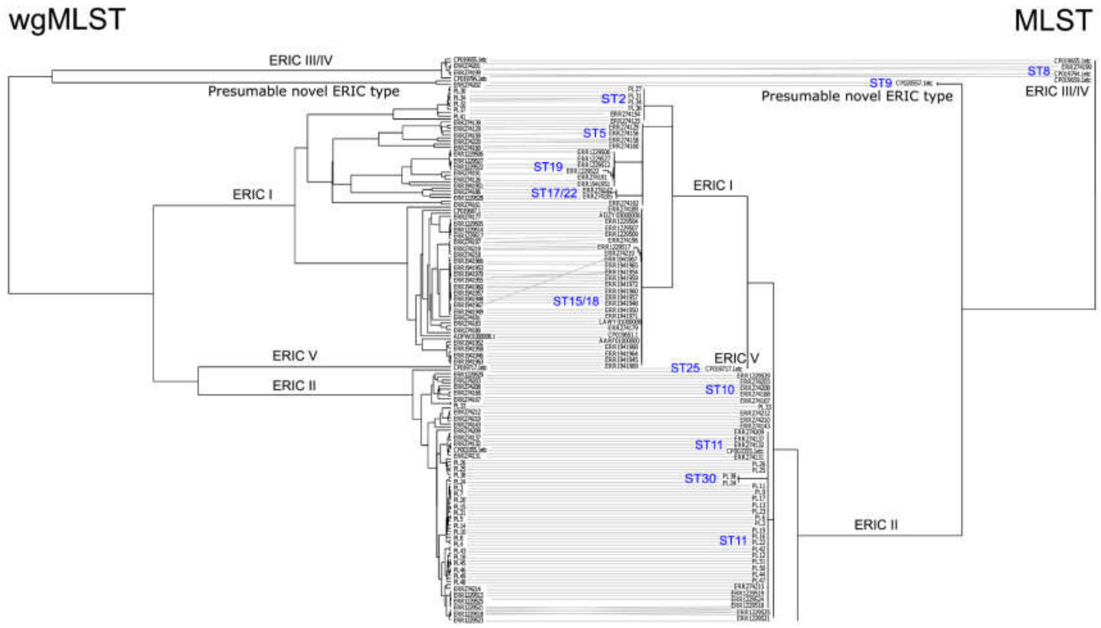


**Supplementary Figure S5**. Comparison of wgMLST and MLST for the complete *Paenibacillus larvae* genome dataset ($n$ = 179). The wgMLST tree was produced in BioNumerics using the categorical (values) similarity coefficient and UPGMA clustering algorithm. For MLST analysis, sequences of seven MLST genes were concatenated and aligned using ClustalW. For the genomes without an assigned ST, all the available MLST sequences were analyzed; these genomes differ to a small extent from the most closely related genomes with an assigned ST. MLST STs are denoted in blue. MLST-based neighbor-joining phylogenetic tree was constructed using Geneious v11.1.5 by applying the Timura-Nei distance model and 100 bootstrap repetitions. Tanglegram of wgMLST and MLST tree was constructed in Dendroscope. Note that some genome labels are omitted from the tanglegram due to spatial limitations. See Supplementary Table S1 for genome metadata.

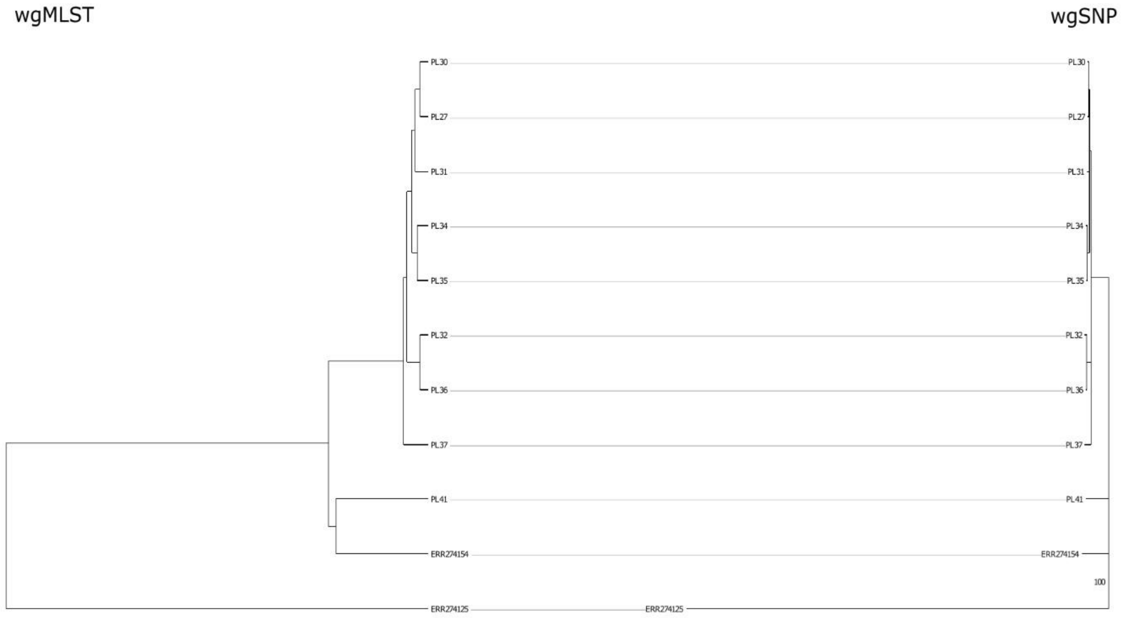


**Supplementary Figure S6.** Comparison of wgMLST and wgSNP for ST2-ERIC I *Paenibacillus larvae* isolates. The wgMLST tree was produced in BioNumerics using the categorical (values) similarity coefficient and UPGMA clustering algorithm. wgSNP analysis was performed in BioNumerics and maximum-likelihood wgSNP phylogenetic tree was inferred using RAxML. Tanglegram of wgMLST tree and wgSNP-based phylogenetic tree was constructed in Dendroscope.

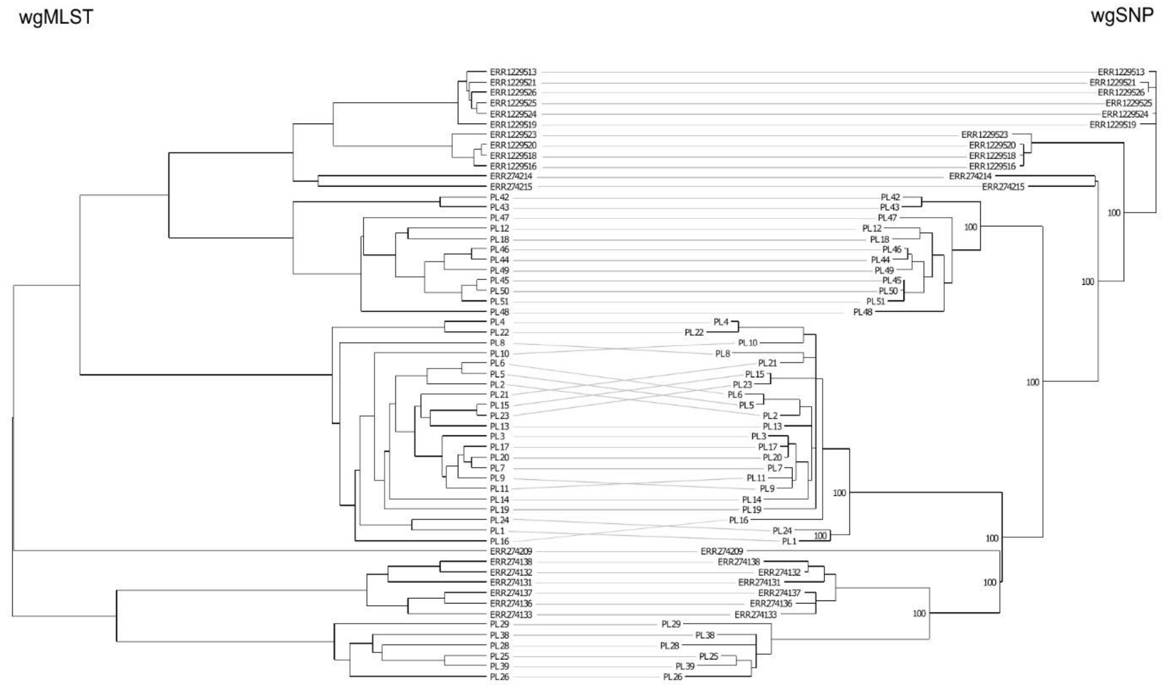


**Supplementary Figure S7.** Comparison of wgMLST and wgSNP for ST11/30-ERIC II *Paenibacillus larvae* isolates. The wgMLST tree was produced in BioNumerics using the categorical (values) similarity coefficient and UPGMA clustering algorithm. wgSNP analysis was performed in BioNumerics and maximum-likelihood wgSNP phylogenetic tree was inferred using RAxML. Tanglegram of wgMLST tree and wgSNP-based phylogenetic tree was constructed in Dendroscope.
