# Supplementary material for: Analysis of the Global Population Structure of Paenibacillus larvae and Outbreak Investigation of American Foulbrood Using a Stable wgMLST Scheme
Source: Front Vet Sci. 2021 Feb 26;8:582677. doi: 10.3389/fvets.2021.582677 (PMC7952629; doi:10.3389/fvets.2021.582677)

frontiers

Supplementary Material

Supplementary File S3 associated with Papić *et al.* – Analysis of the global population structure of *Paenibacillus larvae* and outbreak investigation of American foulbrood using a stable wgMLST scheme

ERIC-PCR typing of the 51 *Paenibacillus larvae* isolates from Slovenia. ERIC patterns were obtained by QIAxcel capillary electrophoresis using the QIAxcel DNA High Resolution Kit with QX Alignment Marker 15–5000 bp and QX Size Marker 100–2500 bp (all Qiagen). PL1–PL51, investigated *P. larvae* isolates obtained from the investigated AFB outbreak in Slovenia. ERIC I, ATCC 9545 reference strain; ERIC II, CCUG 49660A reference strain; ERIC III, LMG 16252 reference strain; ERIC IV, LMG 15974 reference strain. NC, negative control (H_2O). Red arrowhead denotes the band of ~2500 bp differentiating ERIC I and ERIC II type.

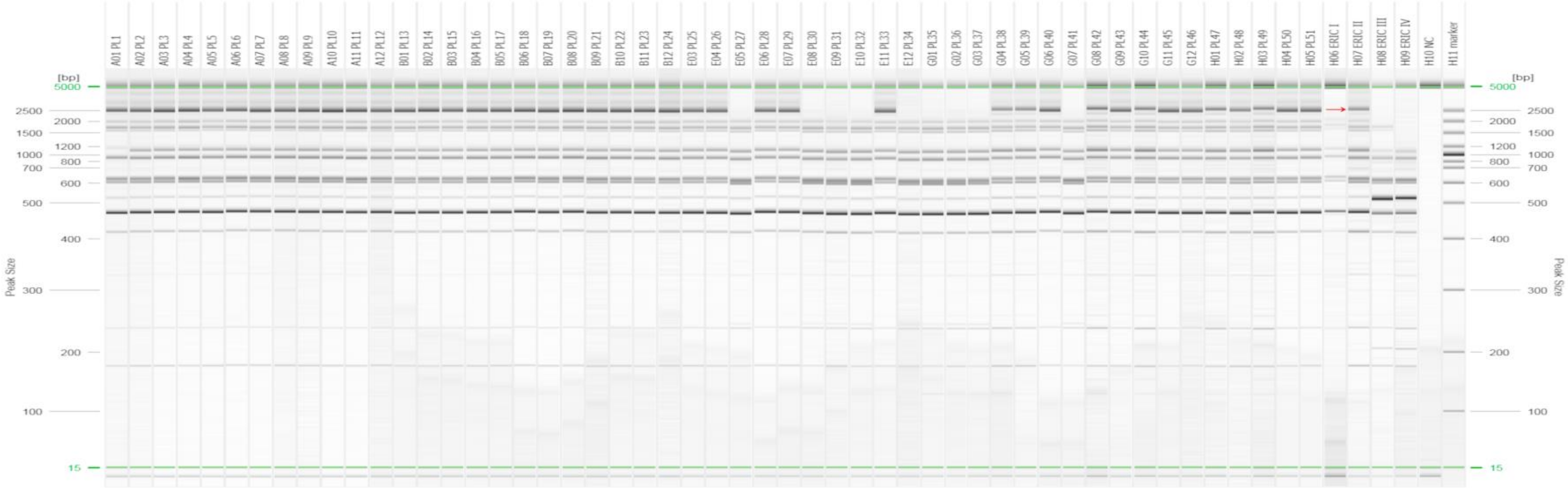

Supplement: Supplementary file 5 [file Data_Sheet_3.PDF]
